# Prevalence of feline leukemia virus infection and associated diseases in a Portuguese domestic cat population: A 4.5-year cross-sectional study

**DOI:** 10.1371/journal.pone.0339172

**Published:** 2026-01-08

**Authors:** Pedro Morais de Almeida, Adriana Belas, Paulo Nogueira, André Meneses, Joana Tavares de Oliveira, Carlos Viegas

**Affiliations:** 1 Faculty of Veterinary Medicine, Lusófona University- Lisbon University Centre, Lisbon, Portugal; 2 Animal and Veterinary Research Center (CECAV), University of Trás-os-Montes and Alto Douro (UTAD), Vila Real, Portugal; 3 Polytechnic Institute of Lusofonia (IPLUSO), School of Health, Protection and Animal Welfare, Lisbon, Portugal; 4 Faculty of Medicine, University of Lisbon, Portugal; 5 I-MVET- Research in Veterinary Medicine, Faculty of Veterinary Medicine, Lusófona University- Lisbon University Centre, Lisbon, Portugal; 6 Animal and Veterinary Research Center (CECAV), Lusófona University- Lisbon University Centre, Lisbon, Portugal; Albany Medical College, UNITED STATES OF AMERICA

## Abstract

In a 2019 Pan-European Study, Portugal exhibited the highest prevalence of Feline Leukemia Virus (FeLV) infection (8.8%). Following the coronavirus disease 2019 (COVID-19) pandemic, it is crucial to evaluate how the prevalence of FeLV has evolved. FeLV infection is associated with the highest morbidity rates, primarily due to the increased incidence of diseases that compromise the health of cat populations, which varies according to the lifestyle and background of the cats studied. This study aimed (1) to estimate the prevalence and temporal trends of FeLV and FIV infections among cats presented to a university veterinary hospital in the Lisbon metropolitan area, and (2) to evaluate the clinical associations between FeLV infection, health status, and FeLV-related conditions in cats. Conducted over 4.5 years, from January 2019 to July 2023, this cross-sectional study took place at a teaching hospital and involved 1,124 cats that were tested serologically and/or by qPCR and RT-qPCR for FeLV. Information was gathered on the intrinsic and extrinsic characteristics of the cats, their health status, and any related diseases. The overall prevalence of FeLV was found to be 11.3% (95% CI: 9.5%−13.3%), with 1.8% (95% CI: 1.1%−2.7%) of cats co-infected with FIV, and it peaked in 2020 at 14.1% (95% CI: 7.5%−23.4%), with 2.4% (95% CI: 0.03%−8.2%) co-infected with FIV. Over the 4.5-year period, an increasing number of cats were tested, and more quantification of proviral and viral loads was performed. This indicated a more progressive course in 47.0% (31/66), of sick FeLV-infected cats, who exhibited a higher incidence of FeLV-related diseases. Although there was no significant difference in the average age between positive and negative cats, FeLV-positive cats demonstrated a higher rate of sickness (74.8%, n = 95). To the best of the authors’ knowledge, this study represents the largest cross-sectional investigation of FeLV infection prevalence and its health implications conducted in Portugal. Overall, the available data suggest a possible increase in FeLV prevalence in Portugal, concurrent with a declining vaccination rate from 14.2% to 5.0%. The results also highlight notable differences in clinical status between progressive and regressive disease courses, reinforcing the necessity of staging the course of infection at diagnosis to ensure an informed medical approach and realistic prognosis. Efforts should focus on improving vaccination and screening activities, promoting neutering of indoor and outdoor cats, and isolating infected cats.

## Introduction

Feline Leukemia Virus (FeLV) and Feline Immunodeficiency Virus (FIV) infections are significant feline health concerns. Understanding the prevalence and factors associated with these retroviral infections is crucial for implementing effective management and prevention strategies. FeLV prevalence varies worldwide, depending on the geographical area and cat population [1–6]. In Europe, southern countries show the highest rates [7–13]. In the 2019 Pan-European Study conducted by the European Advisory Board on Cat Diseases, Portugal displayed the highest prevalence (8.8%) of FeLV infection in a global cat population, based on 330 samples from the Lisbon Metropolitan Area [7]. Subsequently, a study encompassing four European countries, which included 240 Portuguese cats receiving veterinary care in clinics and hospitals within the same demographic area demonstrated a prevalence of 20.4% [14]. Besides these two studies, research on FeLV prevalence in Portugal has been limited to stray cat populations [15–17] and one shelter-based study [18] reporting prevalence rates ranging from 5.7% to 8.5% among stray cats and 5.5% in the shelter population.

Previous studies in various other European countries suggested a stagnation in FeLV prevalence [10,19], a trend also observed in the Australia, Canada, New Zealand and United States [4,6]. During the interim pandemic period of the coronavirus disease-19 (COVID-19), it has become imperative to undertake a comprehensive evaluation of the current situation, particularly in these southern European countries [7].

In addition to geographic location, the likelihood of a cat being or becoming FeLV infected is increased by several factors. These factors include being an intact male aged between one and six years; residing both indoors and outdoors, or exclusively outdoors, living in a group of at least five cats, and having a poor health status [5,6,20–24]. These risk factors are more prevalent in southern Europe, as they are directly linked to the socio-economic and cultural conditions of the population [7,14].

However, in the context of FeLV, additional factors contribute to its persistent status as one of the most substantial infectious diseases. For instance, the methods used to test for FeLV and the complexity of interpreting the results also play a role. Like all retroviruses, FeLV is characterized by a complex pathogenesis that involves a dynamic interplay between the virus and its subgroups, infection pressure and viral load [17,25–27]. Additionally, the immune system and the age of the cat play crucial roles in the eventual integration of the DNA copy of the virus’s RNA genome into the chromosomes of an infected cell [28]. This is a dynamic process that takes several weeks and may change over time, especially with fluctuations in the host’s immune system or the occurrence of re-infections. Four distinct outcomes or courses may arise from the moment the virus comes into contact with the cat through bodily secretions, sexual contact, or blood, such as during fights [23,29,30]. One of these outcomes is the abortive course, where replication occurs only in the oropharyngeal tissue, posing no future consequences for the cat’s health, which acquires antibodies detected by indirect fluorescent antibody assay, but tests negative for the p27 capsid protein detected by point-of-care enzyme-linked immunosorbent assay (PoC ELISA), and PCR tests [31]. Approximately 5% of cats may experience the focal (atypical) course, where their immune system fails to eliminate the virus but can sequester viral replication in specific tissues, such as the spleen and lymph nodes resulting in circulating antigens that yield weakly positive results on PoC ELISA, usually with positive antibody tests but negative PCR results due to a lack of viral or proviral load in circulation [30,32]. In other cats, this may be transient, leading to the regressive course, where the virus integrates into organs and/or the bone marrow DNA (provirus), manifesting discordant PoC ELISA and PCR results, weak viral spread, and low viral and proviral loads. In areas of intense infectious pressure, it is possible for up to 25% of cats exposed to FeLV to develop regressive infections [29]. In these areas, as many as 30% of cats exposed to FeLV may develop a progressive course, where the virus disseminates especially throughout all lymphoid and glandular tissues, and mucous membranes, yielding positive results for PoC ELISA immunofluorescence and PCR testes, with viral loads that tend to be high [4,23,26,27,29]. Recent European studies indicate prevalence rates ranging from 1% to 9% for this condition in cats presented to veterinary care [7,14].

These infection courses are accompanied by distinct, yet also dynamic serological and molecular profiles. This variability is why there is no “gold standard” for reliably diagnosing FeLV throughout the infection course [33–35]. To effectively distinguish between a progressive and a regressive course, an efficient strategy is to repeat testing over time a least eight weeks apart [4,36] employing quantitative p27 antigen and quantitative proviral DNA to classify cats into high positive and low positive courses [26,29,32,33,37]. Distinguishing between these courses is essential for predicting how the infection will affect on the development of FeLV-related diseases and survival times in FeLV-infected cats [25,26]. It is unlikely that cats with regressive infection will develop FeLV-related disease, and most studies support this assertion [23,32,34,38]. These cats may test positive on a single test, often a PoC ELISA test, which can lead to incorrect diagnostic approaches and medical decisions. Two major concerns arise in these cats: in states of immunodeficiency, the provirus may be reactivated and progress to a progressive state. Additionally, the possibility of the proviral DNA present in these cats interacting with cellular proto-oncogenes through somatically acquired insertional mutagenesis remains to be clarified, potentially resulting in neoplasms, even in older cats [28]. The manifestation of clinical and pathological severity conditions has been exclusively observed in cases of progressive infection with cats having 62 times more likely to develop lymphoma or leukemia compared with their noninfected counterparts [39]. Other life-threatening diseases associated with FeLV (or FIV) include blood dyscrasias, leading to anemia, neutropenia, and lymphopenia; increased occurrences of neurological dysfunction; immune-mediated diseases (such as uveitis and polyarthritis); and immunodeficiency, which heightens susceptibility to concurrent infections. Other clinical conditions include chronic gingivostomatitis, reduced serum albumin/globulin ratio (A/G ratio) and hypergammaglobulinemia, reproductive disorders, and fading kitten syndrome [14,22,23,40–44].

The highest probability of developing these conditions is directly linked to a decline in quality of life and a reduction in life expectancy of 3–5 years in cats with FeLV-positive test results [26,28,25, 45-46–]. Studies evaluating the survival of FeLV-positive cats throughout the infection course concluded that only the progressive course/ high-positive status was associated with a decrease in life expectancy, ranging from less than one to three years. In contrast, cats with regressive state (low positive) have a similar life expectancy as those with negative test results [3,26,34,40]. Another critical finding is that, even in progressive infections, several years may pass before FeLV-related diseases manifest and lead to death [34,25].

The prognosis and planning for follow-up in cats with a progressive course may be improved by identifying the specific FeLV subgroups most commonly associated with various FeLV-related conditions [47,48]. The three best-known and most common subgroups are: FeLV-A, which is ubiquitous and more contagious, but less pathogenic [49]; the other two subgroups can result from recombination within this subgroup. The FeLV-B subgroup is less contagious, but is associated with a higher mortality rate due to an increased incidence of lymphoma and leukemia [50,51]. The FeLV-C subgroup is linked to non-regenerative anemia [29,47].

Given the lack of safe and/or effective antiviral therapies [52–54], the best approach to managing this infection is accurate and frequent testing when warranted, along with vaccination whenever a cat has one or more risk factors, such as access to the outdoors or living in an environment with a high density of cats [4,23,29,55]. It has also been found that even vaccinated cats may allow FeLV to integrate a copy of the viral genome DNA into a chromosome of the infected host cell, although vaccination protects against FeLV-related disease and extends life expectancy [56–58]. According to a 2019 report, Portugal had a vaccination rate of 14.25% for FeLV, which is significantly below the European average of 27.1% for vaccinated cats [7].

The primary aim of this study was to consolidate data on the FeLV prevalence and its evolution in relation to demographics, environmental and welfare characteristics in cats taken to a university veterinary hospital in the Lisbon metropolitan area over a period of 4.5 years. A secondary aim was to assess the impact of FeLV infection, with or without co-infection with FIV, on health status, as well its correlation with clinical and clinicopathologic conditions that are directly associated with their FeLV courses, based on viral and proviral loads.

## Materials and methods

### Study design and population

This study is a cross-sectional investigation of all cats that underwent routine veterinary care and were tested for FeLV and FIV for various reasons, including routine screening, vaccination, neutering, or diagnostic testing at the Veterinary Teaching Hospital of the Faculty of Veterinary Medicine – Lusófona University (VTHFVM-UL), located in the Lisbon metropolitan area, during the period from January 2019 to July 2023.

All diagnostic procedures were performed as part of the cats’ medical care during consultations. An informed consent form was signed by the owner, caregiver, or rescuer for the use of data and information for further study. The study followed internationally recognized high standards of veterinary clinical care for each individual patient. The study protocol was reviewed and approved by the Ethics and Animal Welfare Commission (CEBEA) of the Faculty of Veterinary Medicine, Lusófona University (Protocol number 120/2022)

**Fig 1:**
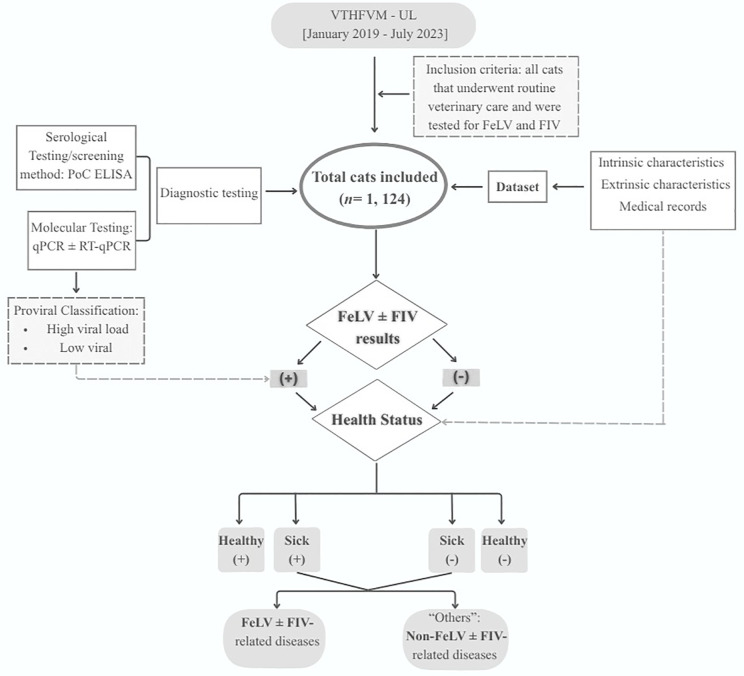
Clinical method for data collection and sample study.

Legend: VTHFVM-UL -Veterinary Teaching Hospital of the Faculty of Veterinary Medicine – Lusófona University; PoC ELISA - point-of-care enzyme-linked immunosorbent assay; RT-qPCR - Reverse transcription quantitative polymerase chain reaction; qPCR-Real-time quantitative polymerase chain reaction; FeLV: feline leukemia virus; FIV: feline immunodeficiency virus; FeLV ± FIV – FeLV and/or FIV positive results. PoC ELISA was used as major secreening method assay to detect FIV antibodies and FeLV antigens. qPCR was performed as a confirmatory test, in referral consultations for retroviruses and FeLV staging based on viral load: high viral load - > 4 × 10⁵ copies/mL (indicative of a “likely” progressive course/ high positive) and low viral load - ≤ 4 × 10⁵ copies/mL (indicative of a “likely” regressive course/ low positive) [26]. Intrinsic characteristics: age, sex, fertile status and breed. Extrinsic characteristics: vaccination status, background, lifestyle (outdoor access), housing conditions and risk cohabitation. FeLV ± FIV-related diseases and conditions: anemia, lymphoma, leukemia and others blood dyscrasias, infections comorbidities and chronic gingivostomatitis; Non-FeLV ± FIV-related diseases also referred to as “others” were categorized by system: respiratory, digestive, urinary, hepatic and dermatological (with the exception of bite abscesses, which were classified as infections), oncological (with the exception of lymphoma and leukemia), systemic infections such as feline infectious peritonitis (FIP) and feline panleukopenia. Multiple trauma, asthma, and lower urinary tract diseases in cats were frequently diagnosed conditions.

A comprehensive database was constructed, encompassing the 1,124 cats tested following the clinical method described in Fig 1.

In terms of data processing, conditions not usually associated to FeLV or FIV were categorized by system: respiratory, digestive, urinary, hepatic and dermatological (with the exception of bite abscesses, which were classified as infections), oncological (with the exception of lymphoma and leukemia), systemic infections such as feline infectious peritonitis (FIP) and feline panleukopenia. Multiple trauma, asthma, and lower urinary tract diseases in cats were frequently diagnosed conditions.

### Data collection

For all cats tested, we began by collating intrinsic characteristics such as age, sex, and reproductive status, along with extrinsic characteristics including vaccination status, environmental influences, access to outdoor environments, and cohabitation with other cats. The selection of these characteristics was based not only on the need to characterize the study population but also on the evaluation of their potential association with the prevalence of retroviral infections. Variables such as age, sex, reproductive status, lifestyle, housing conditions, and medical history have been previously identified as potential risk factors for FeLV and FIV infections [5,10,23,30,45]. For cats with no available information, especially some cats from catteries, rescue facilities, shelters, or stray cats captured in the trap-neuter-return system, their age was estimated based on body size, dentition, and other physical characteristics [59–61]. Cats were classified as junior (≤ 2 years), adult (3–6 years), mature (7–10 years), senior (11–14 years), and geriatric (≥15 years) according to the Life Stage Guidelines of the American Association of Feline Practitioners and American Animal Hospital Association Feline Life Stage Guidelines [62].

### Environmental classification: lifestyle, housing conditions and background

For the purpose of environmental classification, the cat population was divided according to two main criteria: background, lifestyle (outdoor access), housing conditions and risk cohabitation. Regarding de background cats were classified into three groups: (1) client-owned cats; (2) cats from shelters, catteries, or rescue facilities; and (3) stray, free-roaming, or feral cats. For the lifestyle (outdoor access): cats were categorized as having outdoor access or no outdoor access. The latter group included cats housed in shelters, catteries, and rescue centers. Concerning housing conditions groups were established in relation to the number of cohabitants: (1) single cat; (2) cat cohabiting with one other cat; (3) cat cohabiting with two or more cats; (4) stray cats and (5) cats from catteries, rescue facilities, and shelters. Finally, the objective of analyzing risky cohabitation necessitated the establishment of six groups: first, cats that lived alone or whose cohabitants had been tested and were negative for retroviruses and without access to the outdoors; second, cats that cohabited with other FeLV-positive cats. Third, cats that cohabit with FIV-positive test results cats. Fourth, cats that cohabit with positive test results for both FeLV and FIV. Fifth, cats that cohabited with stray, free-roaming and feral cats. Sixth, cats from catteries, rescue facilities, and shelters.

### Diagnostic testing

#### Serological testing.

The primary screening method employed was a point-of-care enzyme-linked immunosorbent (PoC ELISA) (SNAP FIV/FeLV Combo Test® kit (IDEXX Laboratories, Hoofddorp, The Netherlands) on EDTA-anti-coagulated whole blood, following the manufacturer’s instructions. Serological screening was performed immediately after sample collection during clinical consultations.

### Molecular testing

Real-time PCR was used (qPCR) for detection and quantification of proviral load as confirmatory tests, for referral consultations regarding retroviruses, and for FeLV staging (proviral load). Reverse transcription quantitative Polymerase Chain reaction (RT-qPCR) for the detection and quantification of viral load was performed for two primary purposes. Firstly, it was used for risk assessment in multi-cat households. Secondly, it was employed for the pre-assessment of the potential use of antivirals. According to new guidelines and recent studies it has been determined that a high proviral load (qPCR > 4 × 10⁵ copies/mL) is indicative of a “likely” progressive course/ high positive, whereas a low proviral load (qPCR ≤ 4 × 10⁵ copies/mL) is suggestive of a “likely” regressive course/ low positive [26,27,30,63]. As the antibodies were not quantified and given that this was a one-off test in most cats, it is important to note that a single isolated test cannot accurately determine the course of FeLV in a cat. Furthermore, abortive or focal phases were not tracked [26,27,29,64].

Both PCRs were performed on DNA and RNA extracted from EDTA whole blood collected during consultations. DNA and RNA were extracted using the appropriate extraction kits (Nzytech, Lisbon, Portugal) following the manufacturer’s indications. The detection and quantification of FeLV proviral DNA and viral RNA were performed on Rotor-GeneTM-Q (Qiagen, USA) following the manufacturer’s indications of FeLVp dtec-qPCR and FeLV dtec-RT-qPCR Kits (Genetic PCR Solutions™, Spain). All reactions were performed at least in duplicate. All analyses were conducted at the Molecular Biology Laboratory of VTHFVM-UL. PCR processing was carried out on average 24 hours after collection.

### Health Status Classification and Disease Grouping

A correlation was established between the test results and the health status of the cats, as well as the diseases that were diagnosed in the sick cats. From our perspective, the investigation of the diseases present, particularly those typically associated with cats with positive test results for FeLV and/or FIV, proved to be a source of interest. To assess the clinical implications of retroviral positivity, cats were categorized as either healthy or sick, based on clinical records or information obtained during consultation. Medical records were accessed via the Boommed, BVET™, Portugal.

All diagnoses were made following a problem-oriented approach aligned with evidence-based veterinary medicine principles. This approach ensures diagnostic accuracy and consistency across cases. Diseases were classified into six categories, with the first five commonly associated with FeLV and/or FIV infection: anemia, lymphoma, leukemia or other blood dyscrasias, other infectious comorbidities, chronic gingivostomatitis and other diseases and conditions, categorized by organ system: respiratory, digestive, urinary, hepatic, dermatological (excluding abscesses), oncological (excluding lymphoma/leukemia), systemic diseases [feline infectious peritonitis (FIP), feline infectious panleukopenia], and miscellaneous conditions (e.g., polytrauma, undiagnosed illnesses).

While it is impossible to predict when these cats will become infected, we found it interesting from a quality and life expectancy perspective, to compare the average ages of positive and negative cats and of sick and healthy cats, as well as their age and health status. We also find it interesting to compare the age, health status and viral and proviral load in cats where this has been quantified.

### Statistical analysis

Statistical analyses were performed using the Jamovi computer software (version 2.5.3; https://www.jamovi.org). Results for continuous variables are presented as mean ± standard deviation (SD) or the median and percentage, depending on the distribution.

The Shapiro-Wilk test was used to assess the normality of continuous numerical variables. Group comparisons were performed using the unpaired Student’s t-test or ANOVA for normally distributed data, and the Mann-Whitney rank sum or Kruskal-Wallis tests for non-normally distributed data, as appropriate. The ANOVA and Kruskal–Wallis tests were followed by Tukey post-hoc and pairwise comparisons, respectively.

Categorical variables were compared using the Chi-square test or Fisher’s exact test, depending on expected frequencies. Associations between continuous variables were evaluate using. Pearson’s or Spearman’s correlation coefficients based on distribution. A p-value ≤ 0.05 was considered statistically significant.

## Results

Analysis of the Prevalence and Impact of FeLV and FIV Infections in Cats.

### Prevalence

Retroviral infections are a significant health concerns for felines. Understanding the prevalence and factors associated with these infections is crucial for implementing effective management and prevention strategies. This analysis aims to consolidate data on the prevalence, demographic characteristics and welfare-related factors associated with these pathologies over a period of 4.5 years. Additionally, this study aims to assess the impact of retroviral infections on the health status of the feline population under investigation.

### Absolute prevalence of infections

Among the 1,124 cats tested, a relatively high prevalence of infections was observed. The absolute prevalence of FeLV was 11.3% (n = 127) (95% CI: 9.5%−13.3%), of which 1.8% (n = 20) (95% CI: 1.1%−2.7%) were cats with coinfection with FIV, was identified, indicating the need for special attention to cats that may be infected with both viruses simultaneously. Additionally, the absolute prevalence of FIV was 8.5% (n = 96) (95% CI: 7.0%−10.3%). Cats with negative results accounted for 80.2% (n = 901) (95% CI: 77.7%−82.5%).

### Annual trends

From 2019 to 2023, infection prevalence varied annually. A total of 1,124 cats were tested, with the highest number of tests performed in 2022, with a total of 431 cats, surpassing the numbers from 2019, 2020, 2021, and up to July 2023, which had 80, 85, 230, and 298 cats tested, respectively (Table 1). The prevalence rates were highest in 2020, with 14.1% (95% CI: 7.5%−23.4%) of which 2.4% (95% CI: 0.3%−8.2%) were cats with coinfection with FIV, followed by a stabilization at relatively low values in 2023, where the prevalence for FeLV dropped to 8.1% (95% CI: 5.2%−11.7%) of which 2.4% (95% CI: 0.3%−8.2%) were cats with coinfection with FIV.

**Table 1:** Annual distribution and prevalence of feline leukemia virus (FeLV) and feline immunodeficiency virus (FIV) in tested cats (2019–2023).

Year	Cats tested (N)	Result	Cats (n)	Prevalence (%; 95% CI)
2019		Negative	65	81.3 (71.0–89.1)
2019	80	FeLV (+)	5	6.3 (2.1–14.0)
2019		FIV (+)	10	12.5 (6.2–21.8)
2019		FeLV + FIV (+)	0	0.0 (0.0–0.0)
2020		Negative	70	82.4 (72.5–89.7)
2020	85	FeLV (+)	10	11.8 (5.8–20.6)
2020		FIV (+)	3	3.5 (0.7–10.0)
2020		FeLV + FIV (+)	2	2.4 (0.3–8.2)
2021		Negative	175	76.1 (70.0–81.5)
2021	230	FeLV (+)	22	9.6 (6.1–14.1)
2021		FIV (+)	27	11.7 (7.8–16.6)
2021		FeLV + FIV (+)	6	2.6 (1.0–5.6)
2022		Negative	339	78.7 (74.5–82.4)
2022	431	FeLV (+)	50	11.6 (8.7–15.0)
2022		FIV (+)	34	7.9 (5.5–10.9)
2022		FeLV + FIV (+)	8	1.9 (0.6–3.6)
2023		Negative	252	84.6 (80.0–88.5)
2023	298	FeLV (+)	20	6.7 (4.1–10.2)
2023		FIV (+)	22	7.4 (4.7–11.0)
2023		FeLV + FIV (+)	4	1.3 (0.4–3.4)

Legend: FeLV: feline leukemia virus; FIV: feline immunodeficiency virus; FeLV (+): cats with FeLV-positive test results; FIV (+): cats with FIV-positive test results; FeLV + FIV (+): cats with positive test results for both FeLV and FIV; N = total number of cats tested per year; n = number of cats in each result category; CI: confidence interval. Prevalence values are reported as % (95% CI); Statistical analysis: p = 0.136. Screening was performed using a point-of-care enzyme-linked immunosorbent assay (PoC ELISA) with the SNAP FIV/FeLV Combo Test kit. Positive results were confirmed by real-time PCR (qPCR).

### Testing methods

Among the 1,124 cats analyzed, the vast majority (93.2%) were tested using the PoC ELISA, confirming it as the predominant diagnostic method. Combined testing approaches—such as PoC ELISA with qPCR for FeLV, with or without qPCR for FIV, and PoC ELISA with qPCR plus qRT-PCR for FeLV—showed a marked increase in 2022, when 24 tests were performed, accounting for 5.6% of all tests conducted that year. A Chi-square test revealed a statistically significant association between the year and the diagnostic method employed (p = 0.002).

### Proviral load and course of disease

The classification of cats based on proviral load revealed that those with a high proviral load (qPCR ≥ 4 × 10⁵ copies/mL) constituted the majority at 47% (31/66), presenting with a “progressive” profile, of which 2 also had FIV. In contrast, 39.4% (26/66) of cats including 2 with FIV exhibited a proviral load (qPCR ≤ 4 × 10⁵ copies/mL) that indicated a “regressive” profile. Notably, there was a statistically significant association between proviral load and the PoC test results (p < 0.001). In FeLV-positive cats with low proviral load (≤ 4 × 10⁵ copies/mL), 92.3% tested positive for FeLV PoC ELISA, while 7.7% were coinfected with FeLV and FIV. Conversely, in FeLV-positive cats with a high proviral load, 93.5% tested positive for PoC FeLV, while 6.5% had FeLV and FIV coinfection. Among 14% (9/66) of cats with a positive PoC test but no proviral DNA detected, these results may suggest a false positive PoC result or a focal course. In a subset of 33 cats in which both proviral load (qPCR) and viral load or viremia (RT-qPCR) were measured, a comparison revealed a positive correlation, indicating that cats with elevated proviral loads also exhibited higher levels of viremia. Significant differences were noted between proviral load and viremia values, with viremia levels being notably higher (p = 0.004), measuring 61.0 × 107 ± 6.04 × 107 copies/mL and 2.70 × 107 ± 5.72 × 107 copies/mL, respectively. However, no significant association was observed between proviral load and viremia (Fisher’s exact test, p = 0.628), indicating that cats with high proviral load (high positive) do not necessarily exhibit higher viremia.

### Intrinsic characteristics: age, sex, reproductive status

When comparing age and test results, significant differences (p = 0.049) were detected through ANOVA, particularly between FIV-positive cats and the other groups; FIV-positive cats were older, with an average age of 5.46 years (n = 95) compared to other groups, which had average ages of 4.32 years (n = 891) for negative cats, 4.23 years (n = 107) for FeLV-positive cats, and 4.90 years (n = 20) for FeLV and FIV-positive cats. The average age of FeLV-positive and FeLV and FIV-positive cats was similar to that of negative cats, with no significant differences. A Pearson chi-square test demonstrated a significant association (p = 0.035), particularly among FIV-positive cats, who tended to be older. The lack of linearity (p = 0.718) implies that other factors, such as environment or comorbidities, may also influence these associations. Both ANOVA test (p = 0.049) and the nonparametric Kruskal-Wallis test (p < 0.001) indicated that FeLV-positive and FeLV and FIV-positive cats have average ages similar to those of negative cats, with no significant differences. Notably, 44.9% (n = 48) of FeLV-positive cats were found in the junior age subgroups, while 35.5% (n = 38) were in the adult subgroups.

Regarding other intrinsic characteristics, the analysis revealed that 53.7% (n = 604) of the cats were male and 46.1% (n = 518) were female.

Among the FeLV-positive, FIV-positive, and FeLV and FIV-positive groups, 52.3% (n = 56), 74.0% (n = 71), and 84.2% (n = 16) were males, respectively. Likewise, 51.4% (n = 54), 55.2% (n = 53), and 57.9% (n = 11) of cats in these groups were intact. Overall, being a sexually intact male was significantly associated with a higher risk of FeLV, FIV, or FeLV and FIV coinfection (p < 0.001 for both associations). The vast majority of the cats were crossbreeds, accounting for 96.5% (n = 1,085).

### Extrinsic characteristics: Background, lifestyle, housing conditions

In terms of background, more than half of the cats in the sample (52.8%, n = 593) were privately owned, while 16.0% (n = 180) came from shelters, catteries, and rescue facilities, and 26.6% (n = 299) were stray cats. According to lifestyle (outdoor access), 40.8% (n = 459) of the sampled cats had outdoor access, whereas 39.9% (n = 448) did not have the opportunity for contact with other cats outdoors. Only 18.5% (n = 208) were the sole cats in their homes, 15.7% (n = 177) lived with one other cat, and 10.95% (n = 122) resided in multi-cat households (more than 2 cats). The risk of FeLV cohabitation was identified in 7.0% (n = 79) of cases, and 11.6% (n = 131) cohabited with FeLV and/ or FIV-positive cats. Finally, regarding background, 25.7% (n = 289) were stray cats, and 10.2% (n = 115) lived in shelters.

In terms of background, more than half of the cats in the sample (55.3%, n = 593/1072) were privately owned, while 16.8% (n = 180/1072) came from shelters, catteries, and rescue facilities, and 27.9% (n = 299/1072) were stray cats. According to lifestyle (outdoor access), 50.6% (n = 459/907) of the sampled cats had outdoor access, whereas 49.4% (n = 448/907) did not have the opportunity for contact with other cats outdoors. Only 22.8% (n = 208/911) were the sole cats in their homes, 19.4% (n = 177/911) lived with one other cat, and 13.4% (n = 122/911) resided in multi-cat households (more than 2 cats). Finally, 12.6% (n = 115/911) lived in shelters and 31.7% (n = 289/911) were stray cats. However, 10 stray cats were not assigned to this group because they belonged to a closed colony with no access to or contact with outside cats. The risk of FeLV cohabitation was identified in 9.8% (n = 79/808) of cases, and 16.2% (n = 131/808) cohabited with FeLV and/ or FIV-positive cats.

Cross-referencing environmental and lifestyle variables revealed statistically significant associations between age group and all assessed factors (p < 0.001 for all). Younger cats were more frequently found in indoor environments with outdoor access or in colonies. In contrast, senior and geriatric cats were mainly found in indoor environments, often living alone or in shelters with limited outdoor access. Adult and young cats were more likely to cohabit with FeLV and/ or FIV-positive cats (Table 2).

**Table 2:** Association between age groups and environment: background, lifestyle (outdoor access), housing conditions and risk cohabitation.

Variable	Category	Junior (n)	Adult (n)	Mature (n)	Senior (n)	Geriatric (n)	Total (n)	p-value
Background	Client-owned cats	225	217	61	76	13	593	<0.001
	Shelter*	65	67	20	19	9	180	
	Stray**	149	95	30	15	3	299	
Lifestyle (outdoor access)	No	168	164	39	59	17	448	<0.001
	Yes	198	160	56	31	7	459	
Housing Conditions	Indoor alone	67	88	19	27	7	208	<0.001
	With 1 cat	67	72	13	20	5	177	
	≥2 cats	50	39	16	13	3	122	
	Shelter*	43	37	12	14	7	115	
	Stray**	148	91	29	14	1	289	
Risk cohabitation	No risk	76	75	16	21	4	193	<0.001
	With FeLV (+)	31	31	7	6	4	79	
	With FIV (+)	23	13	0	6	1	43	
	With FeLV + FIV (+)	19	46	11	7	5	88	
	Stray**	145	92	29	15	1	288	

Legend: FeLV (+): feline leukemia virus positive results; FIV (+): feline immunodeficiency virus positive results; FeLV + FIV (+): positive results for both FeLV and FIV; n = number of cats per category; N = total number of cats included in the study (N = 1,124). *Shelter: includes cats from shelters, rescue facilities, and catteries; **Stray: includes stray, free-roaming, and feral cats. Cats were classified as junior (≤ 2 years), adult (3–6 years), mature (7–10 years), senior (11–14 years), and geriatric (≥ 15 years) according to the Life Stage Guidelines of the American Association of Feline Practitioners and American Animal Hospital Association Feline Life Stage Guidelines [62]. The table summarizes the results of four chi-square analyses assessing the relationship between age groups, background, lifestyle (outdoor access), housing conditions, and risk cohabitation. For each variable, Pearson’s chi-square test was applied to evaluate the association between categories. All tests revealed statistically significant associations (p < 0.001), indicating that the distribution of age groups varied across environmental and cohabitation contexts.

When analyzing these lifestyle and housing conditions concerning test results for both retroviruses, the findings showed that FeLV-positive cats included 48.6% (n = 52) privately owned and 39.3% (n = 42) stray cats; among cats with outdoor access, 63.9% (n = 62) were identified as FeLV-positive. Only 15.6% (n = 15) lived without cohabitants, and merely 17.15% (n = 14) had no history of risk for FeLV and/or FIV infection. Among FIV-positive cats, 51.0% (n = 49) were stray cats, with 69.9% (n = 65) having outdoor access; only 10.1% (n = 8) had no history of risk for FeLV and/or FIV infection. Of the 20 FeLV and FIV-positive cats, 55% (n = 11) were stray, 80% (n = 16) had outdoor access, and all had a history of risk of infection.

Taken together, the results indicate that intrinsic characteristics—such as male sex and age between 1 and 6 years—and extrinsic factors, particularly outdoor access (p = 0.002), were associated with higher prevalence rates. Similarly elevated prevalence values were observed in cats living in colonies or shelters (p = 0.001).

Vaccination status was the most challenging variable to confirm; only 5.0% (39/779) of cats had up-to-date FeLV vaccinations, six of which were FIV-positive. Among those vaccinated against FeLV, none tested positive for infection, indicating significant effectiveness of vaccination in preventing FeLV (p = 0.034).

### Health status

To analyze the impact of FeLV prevalence on health status, we classified the sample into three groups: healthy cats, sick cats that were FeLV-positive, and sick cats that were FeLV-negative. The results are as follows: cats were found to be healthy at the time of testing in 54.4% (n = 611/1124) of cases, while 45.6% (n = 513/1124) were identified as being ill. Of the sick cats, 18.5% (n = 95/513) were found to be FeLV-positive, whereas 81.5% (n = 418/513) were FeLV-negative.

The mean age for healthy cats was 2.92 years (SE = 0.11, 95% CI: 2.71–3.10); for sick cats that were FeLV-positive, the mean age was 4.52 years (SE = 0.37, 95% CI: 3.79–5.25); and for sick cats that were FeLV-negative, the mean age was 6.51 years (SE = 0.22, 95% CI: 6.07–6.94). The analysis of variance (ANOVA) test revealed significant differences (p < 0.001) in the mean age among the health status groups. The Tukey or adjusted Kruskal-Wallis tests confirmed that sick cats, regardless of FeLV status, are generally older than healthy cats, with sick FeLV-negative cats being the oldest group among the three health states analyzed.

The results gained further significance when examined through the lens of age subclasses (Table 3), indicating that the majority of young cats (juniors and adults) belong to the healthy cat group. Sick cats, particularly FeLV-negative ones, are significantly older. This finding was further validated through the Pearson chi-square test, which revealed a statistically significant association (p < 0.001) between health status and age subclass. The impact of cats infected with the FIV virus and their longevity may be a contributing factor to this finding.

**Table 3:** Distribution of cats by age subclass and health status (N = 1,124).

Age subclass	Healthy/Routine n (%)	FeLV (+) Sick n (%)	FeLV (–) Sick, n (%)	*Total n (%)
Junior	335 (72.2)	36 (7.8)	93 (20.0)	464 (41.7)
Adult	205 (51.8)	40 (10.1)	151 (38.1)	396 (35.6)
Mature	32 (28.3)	8 (7.1)	73 (64.6)	113 (10.2)
Senior	20 (17.9)	6 (5.4)	86 (76.8)	112 (10.1)
Geriatric	8 (28.6)	5 (17.9)	15 (53.6)	28 (2.5)
Total	600 (53.9)	95 (8.5)	418 (37.6)	**1113 (100)

Legend: FeLV: Feline leukemia virus. FeLV (+): cats with FeLV-positive test results; FeLV (-): cats with FeLV-negative test results. Percentages represent the proportion of cats within each age subclass. Cats were classified as junior (≤ 2 years), adult (3–6 years), mature (7–10 years), senior (11–14 years), and geriatric (≥15 years) according to the Life Stage Guidelines of the American Association of Feline Practitioners and American Animal Hospital Association Feline Life Stage Guidelines [62]. *Percentages within each health status subclass are presented horizontally (by row). The “Total” column should be read vertically, indicating the overall percentage of cats in each age subclass; **In 11 of the 1,124 cats classified as belonging to the healthy/routine group, information on age was missing. Pearson’s chi-square test was applied to assess the relationship between health status and age subclass. A statistically significant association was found (χ² = 187, df = 8, p < 0.001).

A significant statistical association (χ2 = 18.383, p = 0.001) was found between the background and health status, with stray cats showing a higher percentage of illness: 13.7% (n = 41/299) unhealthy and tested positive for FeLV, while 42.5% (n = 127) were unhealthy and tested negative. Notably, among the sick cats, one-third were FeLV-positive. A significant association between lifestyle (outdoor access) and disease status was observed (χ² = 17.038, p = 0.002); 13.1% (n = 60/459) of unhealthy cats tested positive for FeLV, while 37.2% (n = 171/459) tested negative.

Regarding the health condition of the infected cats, out of the 1,124 cats tested, 127 were positive for FeLV, with 20 of these also having a coinfection with FIV. Among the FeLV-positive cats, 73.8% (n = 79/107) were sick, while of the 20 FeLV and FIV-positive cats, 16 were ill. In contrast, only 38.6% (n = 348/901) of the negative cats were sick. This indicates that FeLV and FIV-positive cats are significantly more likely to be sick, than negative cats (p < 0.001). It is noteworthy that of the 96 cats that tested positive for FIV, 73% (70/96) were also ill. Interestingly, sick FeLV and/ or FIV-positive cats, on average, tended to be older than both healthy and negative cats.

In FeLV-positive cats where proviral load was quantified (n = 66), those with a high proviral load (progressive) comprised 47.0% (n = 31) and were more frequently ill than those with a low proviral load (regressive), which accounted for 39.5% (n = 26) (χ2 = 57.705, p < 0.001). Only 16.1% of cats with high proviral loads were healthy, and among those that were ill, 80.6% had FeLV-related diseases. Conversely, 42.3% were of cats with low proviral loads were healthy at the time of testing.

### Health conditions and diseases

The most common condition observed in FeLV-positive cats was anemia (17.8%), which was also frequent among FIV-positive (11.5%) and FeLV and FIV-positive cats (20.0%). (Table 4). FeLV-positive cats showed a higher proportion of lymphoma (18.7%), and leukemia and myelodysplastic processes were almost exclusive to FeLV infection (15.9%). Infections and chronic gingivostomatitis were also observed across all groups, but were slightly more common in FIV-positive than in FeLV-positive cats. Among negative cats, infections (6.8%), anemia (3.9%), and chronic gingivostomatitis (2.9%) were the most frequent conditions. Additionally, 23.2% of negative cats presented with various other conditions grouped as “others,” which mainly included trauma, dermatological and respiratory issues, digestive disorders, urinary problems, and oncological diseases.

**Table 4:** Most common conditions seen in cats by retroviral status.

Disease/condition	FeLV (+) N = 107 n (%)	FIV (+) N = 96 n (%)	FeLV + FIV (+) N = 20 n (%)	Negatives N = 901 n (%)
Infection/Immunosuppression	18 (16.8)	17 (17.5)	3 (15.0)	61(6.8)
Anemia	19 (17.8)	11 (11.5)	4 (20.0)	35 (3.9)
Chronic Gingivostomatitis	12 (11.2)	23 (24.0)	2 (10.0)	26 (2.9)
Lymphoma	20 (18.7)	13 (13.5)	1 (5.0)	8 (0.9)
Leukemia/Myelodysplastic Process	17 (15.9)	3 (3.1)	0 (0.0)	4 (0.4)
Other*	23 (21.5)	34 (35.4)	6 (30.0)	209 (23.2)

Legend: FeLV: Feline leukemia virus; FIV: Feline immunodeficiency virus.

FeLV (+): cats with FeLV-positive test results; FIV (+): cats with FIV-positive test results cats; FeLV + FIV (+): cats with positive test results for both FeLV and FIV.

Percentages represent the proportion of cats within each infection group presenting the condition. Cats may present with more than one diagnosis. * Other includes trauma, dermatological issues, digestive disorders, urinary problems, and oncological conditions. Screening was performed using a point-of-care enzyme-linked immunosorbent assay (PoC ELISA) with the SNAP FIV/FeLV Combo Test kit. Positive results were confirmed by real-time PCR (qPCR).

## Discussion

FeLV prevalence varies significantly among countries and regions, depending on the population tested. To the best of our knowledge, only two studies have focused on FeLV infection in cats admitted to veterinary facilities, with approximately a quarter of our sample being from the same geographical area, the Lisbon metropolitan area. One of these studies was the Pan-European study, in which Portugal participated with data from 330 cats [7,14]. Comparing the absolute prevalence obtained, in our study, which was 11.3% of FeLV- positive cats, of which 1.8% had co-infection with FIV, to that recorded in the 2019 Pan-European study (8.8%) showing an overall European prevalence of 2.3%, we find that southern Europe has the highest prevalence rates (5.75–8.8%). Our results indicate that Portugal apparently remains the European country with the highest prevalence of FeLV, surpassed only by those documented in the Middle East, Africa, and Latin America in worldwide seroprevalence studies [1,2]. This asymmetry had already been confirmed in previous studies [9,10,13,20,65] and in subsequent studies conducted after this period in countries such as Italy and Greece [11,12] which reported an overall prevalence of FeLV at 9.6% and 5% respectively. In contrast, countries such as the Netherlands showed a prevalence of 0%, in a study published in 2023, specifically in rural stray cats [66].

Over the years during which this cross-sectional study was conducted, fluctuations in FeLV prevalence were observed, peaking in 2020 at 11.8% for FeLV-positive cats and 2.4% for cats co-infected with FIV. Following this peak, a stabilization was noted, with relatively low values in 2023 of 6.7% and 1.3% for FeLV-positive cats and cats co-infected with FIV, respectively. Although published data on the evolution of FeLV prevalence in cat populations presented to veterinary facilities over the last five years are lacking, trends reported in previous years in countries such as Switzerland, the United States, Canada, Australia, and New Zealand indicated stagnation [4,6,65]. The peak prevalence observed in this study in 2020 may have been due to the more severe period of confinement related to the COVID-19 pandemic. During this period of severe movement restrictions imposed by the Portuguese government, routine veterinary appointments were reduced, and owners had greater difficulty controlling the movements of outdoor-access cats. In addition, some individuals relocated street or community cats they had been feeding into shared spaces without quarantine, and similar situations were reported in shelters and rescue facilities. Although no concrete data are available on the impact of these actions, clinical feedback from that year may help explain the peak in prevalence observed. Our results show that by July 2023, 298 cats had already been tested that year, which is the second-highest number per year, making it is the second highest number tested in a single year, surpassed only by the previous year when 432 cats were tested. Results for PoC ELISA and quantification of proviral and viral load constituted the combined diagnostic method that saw an increase in its use in 2022, representing 5.6% (24/429) of tests performed for the diagnosis and staging of FeLV. This increase aligns with improvements in veterinary care, management, social interaction, and income, as well as enhanced testing methodologies, as analyzed in a previous study [1,7].

During these 4.5 years, the PoC ELISA test predominated in all years, accounting for 93.2% of cases. In 6.3% of cases, results were combined or replaced by viral and or proviral load quantification. A strong agreement between the tests confirmed that viral load is a robust indicator of FeLV infection status, enabling the staging of 39.5% cats as “likely regressive”/ high positive and 47% cats as “likely regressive”/ low positive, particularly in sick cats in which this analysis, combining several methodologies, was performed. Studies that have assessed FeLV prevalence based on the courses of infection have found that the regressive course is more prevalent than the progressive one [14,67,68]. The high prevalence values for both courses, with more cats categorized in the progressive course, may be attributed to the fact that proviral load quantification was mainly performed in sick cats, with FeLV-related diseases and not as a general screening method. This further reinforces the notion that the progressive course is more closely associated with illness than the regressive state.

In terms of factors influencing the prevalence of FeLV infection, both FeLV-positive and FeLV and FIV-positive cats had similar average ages to negative cats, with no significant differences. Notably, cats with higher mean ages were those that tested positive for FIV. Although a survival analysis could not be performed, this finding may simply reflect the chronic nature of FIV infection [4,23,29,69]. Regarding FeLV, only the progressive course negatively impacts life expectancy, reducing it by less than one year to a maximum of three years according to studies focused on survival analyses [3,20,26,34]. Our results may be influenced by the fact that only a minority of cats were staged, and it is plausible that many of these cats are not progressive or have only recently become infected. The duration until cats develop FeLV-related disease can be several years [34]. These data also underscore that advanced age is associated with a higher likelihood of infections, reinforcing the need for careful monitoring in older cats.

Cross-referencing age with other risk factors such as sex, reproductive status, lifestyle, housing conditions, and background, we found that cats exposed to the outdoors or those living in multi-cat housing had a higher prevalence of retroviruses positivity. Young cats were more commonly found in colonies, while adult cats are had greater exposure to cohabitation with FeLV and FIV-positive cats. In contrast, older cats were more frequently found living alone or in shelters (p < 0.001). A significant association was identified between outdoor access and age groups, with younger cats more likely to have access to the outdoors, while elderly and geriatric cats had less access (p < 0.001). A significant association was also noted between risky cohabitation and age groups. Although this study did not assess risk factors, the analysis may suggest that FeLV and FIV infections affect cats in ways that correlate with factors such as age, sex, reproductive status and living conditions. This is consistent with numerous studies indicating a higher risk of FeLV infection among adult intact male cats displaying aggressive behavior, clinical signs, those that are FeLV-unvaccinated, and those with outdoor access or living in multi-cat households, especially with FeLV-positive cats or as strays [7,20,21,24,30,62,70].

Portugal has a substantial population of free-roaming and stray cats, which are considered a synanthropic species left unneutered and valued for their hunting activities [7,71]. Additionally, many owners and shelters do not test their cats and face difficulties in maintaining isolation and preventing FeLV infection [7,14]. This finding reinforces the notion that results from retrovirus prevalence studies, where close contact and population density play a decisive role, are strongly influenced by the background of the studied population, even when located within the same geographical area. For instance, three studies focusing exclusively on stray cat populations [15–17] and one study of a shelter population [18] conducted in Portugal reported prevalence rates between 5.7% and 8.5% for stray cats and 5.5% for the shelter population. The higher prevalence observed in cats receiving veterinary care in our study, where nearly half were client-owned, may be attributed to factors such as a lack of testing upon the adoption of new cats, outdoor access, and insufficient vaccination. These findings further highlight the need for effective care measures.

Regarding factors influencing the course of infection and disease development, our analyzes of the health status of the studied cat sample, revealed that diseases are more prevalent in mature and elderly cats regardless of their FeLV infection status. Most young cats (junior and adult) were grouped in the “Healthy/Routine” category. When comparing the ages of FeLV and FIV-positive cats to those that were negative, it was noted that the former tended to be older or of similar ages, but they exhibited greater complications associated with general health. Specifically, 73.8% of FeLV-positive cats were ill compared to only 38.6% of FeLV-negative sick cats. Our results are consistent with the well-established evidence that both age and FeLV infection are linked to increased morbidity [23]. The older age of the sick FeLV and/ or FIV-positive cats may be due to the fact that almost half of the FeLV-positive cats were client-owned with regular veterinary follow-ups. The higher average age of FIV-positive cats may also indicate a potential correlation with the progression of infection.

Cats categorized as mature, senior, and geriatric categories are more likely to be found in the sick FeLV-negative group with diseases not typically associated with FeLV infection. Although our data did not establish any sex association with specific health status, spayed cats were more likely to be found in the FeLV-negative sick group. This association may be linked to the excess weight commonly observed in neutered cats, as indicated by several published studies on life expectancy and commonly diagnosed disorders in domestic cats [44,72].

Regarding the impact of lifestyle and background on health status, our results indicate that proportionally, the sickest group was the stray cats, one-third of whom were FeLV-positive, while pet cats with outdoor access ranked among the sickest. This aligns with feline well-being recommendations advocating for keeping pet cats indoors [4,23,73]. The analysis also underscored that cohabitation and living conditions directly impact cat health, suggesting that “protected” conditions—such as living without outdoor access—are associated with better health indices.

Among the cats for which viral and proviral loads were measured, those with a high viral load (progressive) were found to be more frequently ill (31 cases) compared to those with a low viral load (regressive), which totaled 26 cases (p < 0.001). Our results support the notion that progressive FeLV cats exhibit a higher prevalence of disease than regressive cats [3,14,22,26,34]. This reinforces the idea that proviral load quantification is an important parameter for prognostic assessment and clinical management.

Although no association has been identified between increased proviral load and increased viremia, further research in this area is warranted due to the limited existing studies. Effective isolation of constant virus shedders from uninfected cats is crucial to prevent transmission; therefore, quantifying proviral load could potentially be used to assess the risk of contagion.

Lymphoma was found to be more prevalent among FeLV and FIV-positive cats, with rates of up to 18.7% and 13.5%, respectively. Alongside blood dyscrasias (specific to FeLV), and leukemia. These results concerning leukemia and lymphomas are consistent with previous studies [6,10,22,41,42,46,74]. Regarding to anemia and other infections, it is important to note that most of the cats in the sample had outdoor access, came from shelters, or were strays. Recent consensus statements suggest that there is limited evidence that FeLV itself induces immune-mediated hemolytic anemia [75] and this lifestyle and background may increase the likelihood of other coexisting infectious diseases [7,23,29].

Our data demonstrates a statistically significant association between vaccination status and test results, supporting the idea that vaccination may confer protection against FeLV infections. However, this study indicates, alongside the increase in FeLV prevalence, an apparent decline in the vaccination rate to 5.0%, compared to the 14.25% recorded in the 2019 pan-European study [7]. These findings emphasize the importance of vaccination as a vital preventive measure. Continuous monitoring combined with appropriate veterinary practices is essential for improving overall feline health.

The primary limitation of the present study was that the data were based on a one-off test; most of the cats were not retested over time. This is relevant because the infection process is dynamic and may evolve over several weeks, with possible changes in infection status related to host immune response or re-infection events. Diagnosis was primarily serological, with proviral load and course of infection being determined in only a minority of cases. It would have been beneficial to quantify anti-FeLV antibodies to screen for abortive and some focal and regressive courses with very low proviral and viral loads. Such data would have enabled a more comprehensive characterization of the various courses of infection within the studied sample. Future investigations should aim for longitudinal, prospective studies for a better assessment of survival analysis regarding FeLV infection, covering the entire geographical area of the country for a thorough characterization.

This study highlights the need to revolutionize the approach to FeLV in terms of diagnosis, monitoring and prognosis. Complete staging is essential, and proviral load quantification should preferably be included for all ELISA-positive cats, while acknowledging that this approach will miss regressive infections. This is particularly important for cats with a progressive course of infection, the only course shown to significantly impact average life expectancy and health status due to FeLV-related diseases [3,4,26,34]. Moreover, characterizing FeLV subgroups may be useful in predicting disease progression associated with infection status in progressive cats. These findings should be published in prospective studies. Genetic characterization of FeLV has already been performed in a population of stray cats in Portugal by the authors [17].

## Conclusion

This study, the largest cross-sectional assessment of FeLV infection in cats receiving veterinary care in the Lisbon metropolitan area, reveals a high and apparently increasing prevalence of FeLV infection in Portugal. The progressive course of infection is clearly associated with morbidity and FeLV-related diseases. These findings reinforce the imperative of vaccination—particularly for cats with outdoor access, stray cats included—along with improved neutering programs, regular screening, and isolation of infected cats to mitigate viral spread. Future longitudinal studies with survival analysis are warranted to further elucidate the temporal dynamics and clinical outcomes of FeLV infection.

## Supporting information

S1 File Data Set. [Sheet 1: Fully anonymised dataset containing demographic, clinical, epidemiological and diagnostic variables for the 1,124 cats included in the study. Sheet 2: Variable key with coding definitions for all recorded parameters.].
(XLSX)
